# PERMA well-being and innovative work behaviour : A systematic literature review

**DOI:** 10.12688/f1000research.141629.1

**Published:** 2023-10-16

**Authors:** Nor Fauziana Ibrahim, Sabri Mohamad Sharif, Hasan Saleh, Nor Hasliza Mat Hasan, Nur Faezah Jayiddin

**Affiliations:** 1 Faculty of Business, Multimedia University, Malacca, Malacca, Malaysia; 2 Technical University of Malaysia Malacca, Durian Tunggal, Malacca, Malaysia

**Keywords:** Systematic Literature Review, Innovative Work Behaviour, PERMA, Well-being

## Abstract

**Background:** The purpose of this research is to examine at how the literature measures the relationship between PERMA (positive emotion, engagement, relationships, meaning, and accomplishments) well-being and innovative work behaviour (IWB).

**Methods:** This systematic literature review examines peer-reviewed English research papers published in 2012 that investigate the relationship between PERMA well-being and IWB. A total of 37 publications were discovered in 32 journals.

**Results:** A total of 220 articles were initially retrieved from the database. 37 studies out of 220 satisfied the inclusion criteria and were thoroughly examined. Our findings present a comprehensive overview of the types of PERMA well-being related to IWB in different countries and industries. Literature-based research approaches are also discussed. Research methods from the previous literature are also discussed.

**Conclusions:** This study is one of the first to conduct a systematic literature review (PRISMA) method on the relationship between PERMA well-being and IWB. This review suggests constructive future research directions.

## Introduction

Innovation is vital to stay competitive in the global market. At the core of every innovation are creative ideas, and it is the individual employees who, either independently or collaboratively, create, promote, discuss, refine, and realise ideas. ^1^ Therefore, it is no surprise that innovative work behaviour (IWB) employees are vital for modern contemporary organizations. The management must be able continuously to enable IWB among their employees especially as the business environment becomes more dynamic. To better understand IWB, both academics and corporate managers have focused on the antecedents of employees’ IWB and how to promote and support employees’ innovativeness. ^2^

Some of the proposed antecedents of IWB include leadership styles and organisation citizenship behaviours, ^3^^,^^4^ job characteristics, job design, organisational support, ^5^^,^^6^ personality, trust, and justice. ^7^^,^^8^ Other studies view the human resource (HR) system and organizational structure as important determinants of IWB. ^9^ Moreover, recent research has highlighted work engagement in shaping employee IWB. ^10^^–^^13^

Although existing research has explored various factors that contribute to IWB, it is necessary to explore the underlying psychological factors involved in positive well-being especially specifying how individual positive well-being impacts IWB. ^14^ Focusing on the positive aspects of employee well-being is likely to result in increased work engagement, which is particularly beneficial for fostering innovation within organizations. ^15^ Employees who are happy and possess a positive mindset tend to demonstrate greater competence, creativity, and actively generating innovative ideas. ^16^^,^^17^

Gaining an understanding of the relationship between employee well-being and IWB is crucial in order to determine the appropriate ways to support and nurture innovative employees. Past studies have focused on examining the influence of individual well-being on the IWB. ^18^^,^^19^

While scholars in the social sciences have increasingly focused on employee well-being, it has been acknowledged that the dimension of employee well-being has been limited to one or two models or approaches ^18^^,^^19^ which cannot represent overall employee well-being. ^20^ Therefore, the main aim of this review is to examine how positive well-being factors influence IWB based workers on the selected literature which covers various model and approaches.

### Theoretical background

Well-being encompasses an individual's holistic experience of their physical, mental, emotional and their level of happiness. At work, well-being is crucial because it greatly influences an individual's physical, mental, and emotional health. ^21^ According to Ogbonnaya and Messersmith, ^22^ if an employee's well-being is compromised, it can lead to reduced productivity, higher absenteeism, increased stress, and negatively affect their work performance and the organisation's overall success. Indeed, positive well-being among employee provides positive impact towards innovative work behaviour. An employee who is physically and mentally healthy and satisfied with their work are more likely to be engaged and motivated to create up creative ideas and solutions. ^23^

IWB refers to creative and unconventional ways of thinking and approaching work tasks and challenges. ^24^ Encouraging employees to engage in innovative work behaviour can lead to increased productivity and improved problem-solving skills, which can in turn contribute to the success of the organization. Following Onne Janssen, ^25^ our understanding of IWB in the workplace involves recognizing it as a multifaceted behaviour comprising three distinct behavioural tasks: generating ideas, promoting ideas, and implementing ideas. Idea generation is the process of creating, developing, and generating new ideas, concepts, or solutions to problems. ^9^ The following step in the innovation process involves promoting ideas to a potential group. Idea promotion occurs once an employee has generated an idea, involving the process of presenting and persuading allies to gain their support and approval for the idea. ^26^ Idea generation and idea promotion can be critical to the success of innovation, as it involves persuading others to invest in or implement the idea, which can help to bring it to market or bring it to life. The last stage will be idea realization by bringing an idea to fruition, from conception to implementation. ^15^^,^^27^^,^^28^

Many research has shown that well-being is connected to IWB. ^4^^,^^17^^,^^29^^–^^32^ However, many studies pointed out that well-being cannot be defined by a dimension measurement. ^1^^,^^18^^,^^33^^,^^34^ Seligman ^34^ argued that well-being cannot be defined by a single measurement dimension, but rather encompasses multiple aspects that can be more easily measured. Emotion, relationship, meaningfulness, achievement and life satisfaction are all highly influenced by an employee’s current mood and situation. In reality, research has demonstrated that only multidimensional measurements of well-being are effective. ^35^ Furthermore, reducing measurement of well-being dimension to single dimension obscures potential vital information according to Morgan and Simmons. ^36^ Psychology experts believe that well-being is best understood as comprehensive set of indicators by covering multiple dimensions rather than as a single element. ^1^^,^^18^ Ascenso ^20^ emphasises heavily on research efforts directed to the development of creative, innovative, valid, reliable, and affordable survey questions for measuring the multiple dimensions of positive well-being.

Seligman ^34^ introduced the PERMA model with five core elements of psychological well-being knowing as “positive emotion” (happiness, joy, pleasure, satisfaction), “engagement” (involvement in a particular task, activity, or relationship), “relationships” (having positive connection, association, or bond with others), “meaning” (sense of purpose, mindful, significance, and coherence in one's life.), and “accomplishment” (regularly achieving successes. ^18^^,^^37^ The PERMA model is widely recognized as a prominent framework for well-being, leading to the rapid adoption of the PERMA Profiler since its publication in 2016. ^38^ In fact, it has been identified that there are five pathways that are considered the most effective in determining what individuals seek for their own well-being, serving as indicators of positive emotions and optimal functioning. ^39^

The PERMA encompasses both hedonic and eudaimonic perspectives of well-being, has been developed. One of the primary critiques levelled against the PERMA model pertains to the limited empirical evidence supporting its claims. ^35^^,^^39^ Recent efforts aimed at bridging this gap supported PERMA's proposed facets of well-being, by using a study sample of over 15,000 individuals from various regions worldwide. ^18^^,^^35^ Current authors consider PERMA model to compliment other unidimensional well-being theories to provide a deeper level of understanding in employee positive well-being. Hence, the aim of this study is to investigate the possibility of measuring the individual constructs of PERMA as distinct dimensions to measure well-being and measure relationship between IWB.

The current systematic literature review aims to answer the following question:

How is the relationship between PERMA and IWB measured?

Specifically, within the broader research question, there were six guiding questions that directed the review:

Q1. What specific methods have been employed in previous studies?

Q2. Which countries/regions have been examined or included in the existing literature?

Q3. Which academic journals are scholars publishing those articles?

Q4. What is the pattern observed or trend of this line of research?

Q5. What are the research samples included in the existing literature?

Q6. What is PERMA well-being? Which specific PERMA factors have been identified as predictors of IWB in the literature? Conversely, which PERMA factors have received comparatively less research attention? What are the reasons for this disparity?

## Methods

### Study design

This systematic literature review had a specific objective of determining how multidimension PERMA well-being affects IWB by examining the current body of literature. This review follows the reporting criteria of Preferred Reporting Items for Systematic Reviews and Meta Analysis (PRISMA) guidelines.

***Eligibility criteria***

Since the objective of this study was to measure PERMA well-being constructs that includes significant relationship with the IWB, we performed a systematic literature review. This technique allows for in-depth analysis of all important publications on this topic, thus improving the quality of the review process and findings. ^9^ We reviewed current theoretical and empirical studies related to well-being and IWB in various industries. Only papers published in peer-reviewed publications beginning in 2012 and written in English were evaluated for inclusion in the review. Studies classified as editorials, comments, opinion articles, or without an abstract were not considered further.

***Search strategy***

We selected five major social sciences databases which were Science Direct, Emerald, ProQuest, Scopus and Springer. Next, with the research focusing on the relationship between well-being and IWB, we employed the Boolean approach and selected the terms 'well-being,' 'innovative work behavior,' and 'PERMA' as the primary keywords for our initial article search. The search process was continued until no new studies meeting the selection criteria were identified.

**Table 1.** Search Strategy.

Innovative Work Behaviour (IWB)	Innovative Work Behaviour (IWB)	Innovative Work Behaviour (IWB)	Data Search
	Well-being	Well-being	1 November to 31 Dec 2022
		PERMA	1 November to 31 Dec 2022

***Extracting and synthesising data***

**Figure 1.**
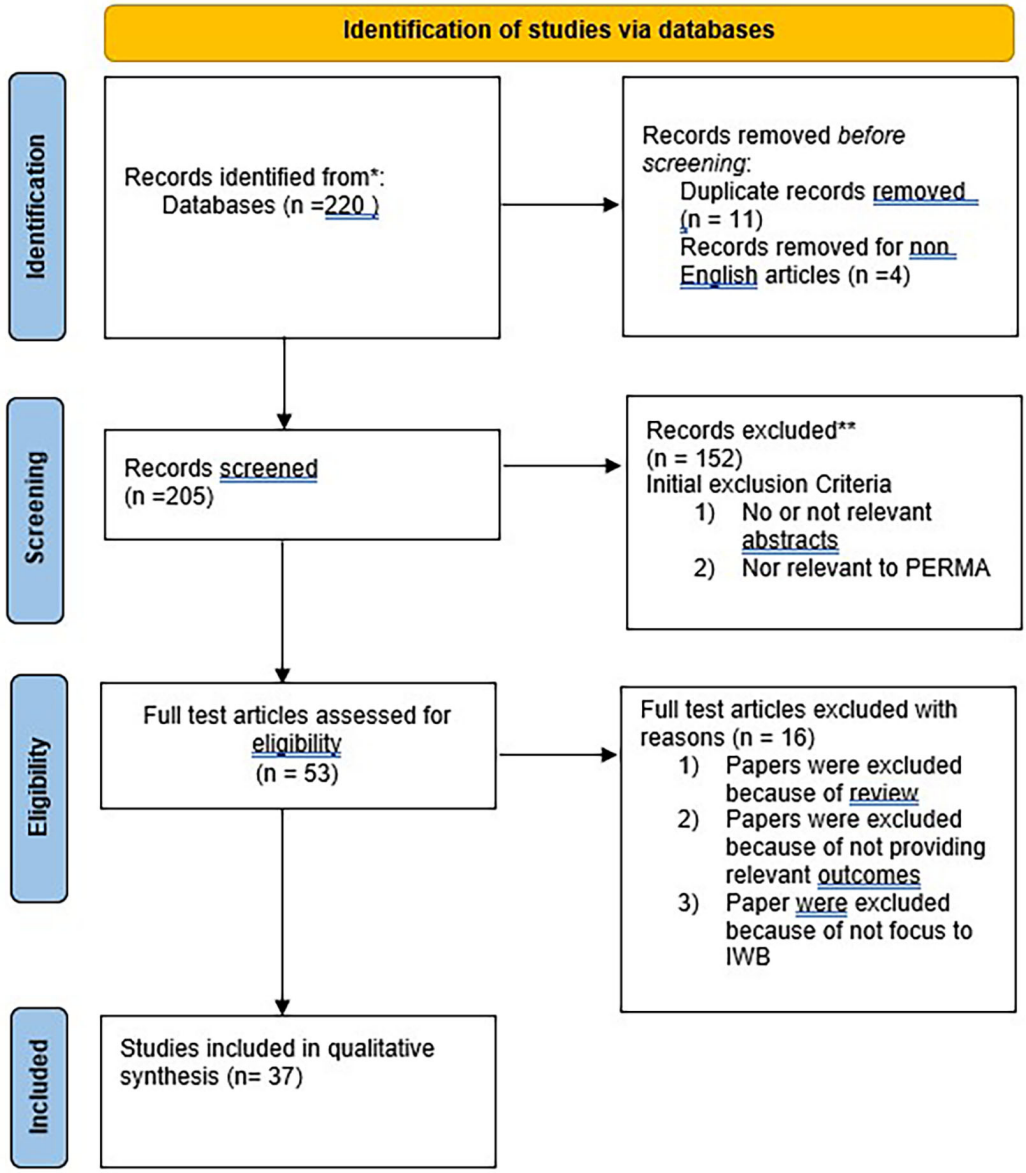
PRISMA flow chart visualizing the article selection process.

Initially, a search in the first trial database yielded a total of 220 results. After removing 15 duplicate and non-English articles, 205 articles remained. The titles and abstracts of these 205 papers underwent screening, leading to the exclusion of 152 papers. This was based on the initial exclusion criteria such as lack of relevant abstracts or irrelevance to PERMA dimensions. A total of 53 papers were selected to underwent a thorough review to assess the eligibility. In order to obtain the final set of papers, further selection and refinement processes were carried out. We excluded 16 papers for the following reasons: they were literature reviews, not solely focused on IWB, and lacked a direct discussion on the significant relationship between PERMA well-being and IWB. Finally, 37 papers were used in a qualitative synthesis. To answer our main research question about the relationship between PERMA well-being and IWB, we analysed 37 articles and extracted relevant information such as publication details, authors, methodology, PERMA well-being dimensions, instruments used, and the findings. This helped us to understand the types of PERMA well-being associated with IWB and how scholars examined them. Figure 1 depicts the PRISMA selection flow process.

## Results

Upon thorough review and analysis of the articles, the results section of this study has been structured into six categories based on the research questions. It encompassed research design, journal titles, publication volume, research samples, geographical distribution of publications, and the PERMA factors of well-being as stated in Table 2.

**Table 2.** Literature review matrix.

Publication Year	Authors	Region/Industry	Methodology/Methods	PERMA Well-Being	Instruments	Journal Titles	Findings
2013	Yesil, S., & Sozbilir, F	Turkey/Service industry (Hotel industry)	Quantitative/PLS-Graph (build 1126), a Partial Least Squares (PLS) Structural Equation Modelling (SEM) tool	Positive Emotion	Personality items: John et al., (2008). Innovation behaviour item: Hu et al., (2009), based on work of Grey & Garrett (2004) and Scott & Bruce (1994)	*Procedia Social and Behavioral Sciences*	Extraversions are not related to individual innovation behaviour.
2015	Krog, C. L., & Govender, K.	South africa/IT industry	Quantitative/Correlation and Descriptive statistics	Relationship	Positive relationship leader: Barbuto and Wheeler (2006)	*SA Journal of Human Resource Management*	Leaders who are communicating pursuasively has the strongest impact on employee innovative behaviour
2015	Mokhber, M., bin Wan Ismail, W. K., & Vakilbashi, A	Iran/Not specified	Quantitative/SEM	Relationship	Multifactor Leadership Questionnaire (MLQ) Form 5X (Bass & Avolio, 1997)	*Iranian Journal of Management Studies (IJMS)*	Positive environment provided by leaders supports innovative teams and organizational innovation.
2015	Abid, G., Zahra, I., & Ahmed, A.	Pakistan/Manufacturing industry	Quantitative/Hierarchical linear modeling	Positive Emotion	Positive Emotion at Work: Porath et al., (2012) Innovative Work Behavior: Scott and Bruce (1994)	*Pakistan Journal of Commerce and Social Sciences*	Thriving at work is positively associated with innovative work behavior.
2016	Akram, T., Lei, S., & Haider, M. J.	China/IT industry	Quantitative/SEM	Relationship	Relational Leadership: Carifio (2010) IWB: Janssen (2000)	*Arab Economic and Business Journal*	Relational leadership is positively and significantly affects the overall EIWB.
2017	Kim, W., & Park, J.	South Korea/Not specified	Quantitative/SEM	Engagement	Work Engagement: Utrecht Work Engagement Scale (Schaufeli et al., 2002)	*Sustainability*	The results show that work engagement and innovative work behavior are positively and statistically significant.
2017	Yildiz, B., Uzun, S., & Coşkun, S. S.	Turkey/Manufacturing industry (White Goods Industry)	Quantitative/Correlation and Descriptive statistics	Relationship	Perceived Organizational Support: Eisenberger, Cunnings, Aemeli, & Lynch, 1997)	*International Journal of Organizational Leadership*	Positive relationship between organizational support and innovative behaviors.
2018	Riaz, S., Xu, Y., & Hussain, S.	China/Mixed industries (Information technology sector, service sector, and manufacturing sector)	Quantitative/SEM-PLS approach	Positive Emotion	Positive Emotion: Porath et al., (2012). Innovative Behavior Scale: Scott and Bruce (1994)	*Adminstrative Science*	Thriving has positive relationship with IWB. Organisation support has positive relationship with IWB.
2019	Aldahdouh, T. Z., Korhonen, V., & Nokelainen, P.	Finland/Education industry	Quantitative/Bayesian Multilevel Path Analysis	Engagement Relationship	Engagement: Midgley and colleagues' (2000) Achievement Goal Orientation (AGO) Scale Supportive relationship culture: Cameron and Quinn (2006)	*International Journal of Innovation Studies*	Mastery goal orentation is positively associated with individual innovativeness. The supportive relationship culture appeared to have neither direct effects on individual innovativeness nor a moderation effect on the relationships between the psychological variables and innovativeness.
2019	Laguna, M., Walachowska, K., Gorgievski-Duijvesteijn, M. J., & Moriano, J. A.	European Union Countries (Netherlands), Poland, and Spain/Not specified	Quantitative/Correlation and Descriptive statistics	Engagement Relationship	Work Engagement: Utrecht Work Engagement Scale (Salanova et al., 2000) (Schaufeli et al., 2002) Authentic Leader relationship: Questionnaire Walumbwa et al. (2008)	*International Journal of Environmental Research and Public Health*	Employee work engagement positively related to innovative behaviour. Work engagement serves as a mediator between authentic leadership and the innovative behaviour of employees. Positive and authentic leader relationship predicts personal initiative and the work engagement of employees, and these variables in turn predict employees’ innovative behaviour.
2019	Saether, E. A.	European Union Countries (Norway)/Technology industry	Quantitative/SEM	Meaningful Positive Emotion	Meaningful (Identified Motivation): Multidimensional Work Motivation Scale by Gagné et al. (2015) Positive emotion: Lauver and Kristof-Brown's (2001) IWB: De Jong and Den Hartog's (2010)	*Journal of High Technology Management Research*	The result indicate that identified motivation is positively related to innovative work behaviour. Intrinsic work motivation is positively related to IWB.
2019	Pandey, A., Gupta, V., & Gupta, R. K.	India/Mixed industries (Manufacturing, banking, telecommunication,and information technology)	Quantitative/SEM	Meaningful	Meaningful (Team-Level Spiritual Climate): Direct-consensus, composition model used by Chan (1998)	*IIMB Management Review*	The findings reveal that spiritual climate is positively related to team-level innovative behaviours.
2019	Opoku, M. A., Choi, S. B., & Kang, S. W.	Ghana/Manufacturing industry	Quantitative/Correlation and Descriptive statistics	Relationship	Positive relationship leader: Liden et al. (2015)	*Sustainability*	Positive Leader characteristic such as servant style facilities IWB.
2019	Odoardi, C., Battistelli, A., Montani, F., & Peiró, J. M.	European Union Countries (Italy)/Mixed industries (Pharmaceuticals, Manufacturing, IT)	Quantitative/Correlation and Descriptive statistics	Relationship	Participative leadership: Arnold et al. (2000)	*Revista de Psicología del Trabajo y de las Organizaciones*	Participative working environment supports employee innovation.
2019	Bin Saeed, B., Afsar, B., Shahjeha, A., & Imad Shah, S	China/IT industry	Quantitative/Correlation and Descriptive statistics	Relationship	Multifactor Leadership Questionnaire (MLQ): Bass & Avolio (1997)	*Economic research-Ekonomska Istraživanja*	The relationship of leader and employees does influence IWB.
2019	Abbas, W., & Wu, W.	Pakistan/Service industry	Quantitative/PROCESS macro	Relationship	NA	*Human Systems Management*	Humility leader relationship is a positive factor that increases innovative behaviors.
2020	Gemeda, H. K., & Lee, J.	South Korea/IT industry	Quantitative/Multiple linear regression analysis and Descriptive statistics	Engagement Relationship	Engagement: UWES-9 (Schaufeli, Martínez, Pinto, Salanova, & Bakker, 2002; Schaufeli, Salanova, et al., 2002) and subsequently reviewed by Schaufeli et al. (2006). Multi-Factor Leadership Questionnaire (MLQ-5X): Avolio et al.(1999) IWB: Janssen (2000)	*Heliyon*	Work engagement had significant positive relationships with innovative work behavior. The relationship between transformational leadership and professionals’ innovative work behavior was partially mediated by work engagement in both countries. The leadership style had significant positive relationships with employees' work engagement and innovative work behavior.
2020	Kundu, S. C., Kumar, S., & Lata, K.	India/Other industry (Corporate sector)	Quantitative/Multiple Regressions and Bootstrapping via PROCESS	Engagement	Intrinsic motivation: Zhang and Bartol (2010) Job involvement: Gazzoli, Hancer, and Park (2012)	*RAUSP Management Journal*	There is a significant positive relationship between intrinsic motivation to innovative work behaviour. Findings also highlight that highly involved employees eagerly participate in their jobs, enabling them to behave creatively.
2020	Pukkeeree, P., Na-Nan, K., & Wongsuwan, N.	Thailand/Not specified	Quantitative/Descriptive Statistic and PROCESS Macro	Engagement Positive Emotion	Employee Engagement: Saks (2006) IWB: Janssen (2000)	*Journal of open Innovation*	Employee engagement positively affected innovative work behaviour. Positive emotion will promote employee engagement (EE). A high level of employee engagement (EE) increases innovative work behaviour (IWB).
2020	Su, W., Lyu, B., Chen, H., & Zhang, Y.	China/Technology industry	Quantitative/Correlation and Descriptive statistics	Relationship	Supportative leader relationship: Liden et al.’s (2015)	*Baltic Journal of Management.*	The results confirm that support and encourgement from leaders can promote employees’ service innovative behavior and intrinsic motivation.
2020	Mutonyi, B. R., Slåtten, T., & Lien, G.	European Union Countries (Norway)/Service industry	Quantitative/SEM	Relationship	Empowering Leaders: Amundsen and Martinsen (2014)	*International Journal of Public Leadership.*	Empowering leaders and individual learning orientation had significant direct effects on individual innovative behaviour.
2020	Jan, G., & Zainal, S. R. M.	Pakistan/Service industry (Hotel and Tourism)	Quantitative/SEM	Relationship	NA	*Asian Academy of Management Journal*	Cooperative work environement exhibit IWB.
2020	Zeng, J., & Xu, G	China/Education industry	Quantitative/Correlation and Descriptive statistics	Relationship	Servant leader relationship: Sun and Wang (2010)	*International Journal of Environmental Research and Public Health*	Servant type leader relationship had a significantly positive impact on innovation behavior.
2021	Dixit, A., & Upadhyay, Y.	India/Education industry	Quantitative/PLS-SEM	Engagement Accomplishment	Employee engagement: UWES–Utrecht Work Engagement Scale (Schaufeli & Bakker, 2004) IWB: Janssen (2000) Reward and recognition: Spector (1985)	*RAUSP Management Journal*	Employee engagement was positively and significantly related to innovative work behaviour. Reward and recognition were significantly related to employee engagement whereas they were insignificant to IWB. Reward and recognition do not impact innovative work behaviour directly, rather, their effect moves through employee engagement.
2021	Ganji, S. F. G., Rahimnia, F., Ahanchian, M. R., & Syed, J.	Iran/Education industry	Quantitative/SEM	Engagement	Work Engagement: Gatenby et al. (2009).	*Iranian Journal of Management Studies (IJMS)*	Employee engagement positively influence innovative behaviour and predict idea generation, idea promotion and idea implementation positively.
2021	Sudibjo, N., & Prameswari, R. K	Indonesia/Education industry	Quantitative	Relationship	NA	*Heliyon*	There is negative effect on leadership relationship and innovative work behaviour (IWB) found in this study.
2021	Grošelj, M., Černe, M., Penger, S., & Grah, B	Not specified/Technology industry	Mixed-method research/Correlation and Descriptive statistics	Relationship	Authentic relationship leader: Neider and Schriesheim (2011)	*European Journal of Innovation Management.*	The research provides further confirmation of the positive relationship between authentic leaders and innovative work behaviour.
2021	Gao, Y., & Liu, H.	China/Technology industry (Science and technologies)	Quantitative/Correlation and Descriptive statistics	Relationship	Supervisor– subordinate guanxi (SSG): Law et al. (2000)	*Psychology Research and Behavior Management*	Supervisor– subordinate guanxi (SSG) was positively associated with employee Innovative behaviour (IB).
2021	González-González, T., & García-Almeida, D. J.	European Union Countries (Spain)/Service industry (Hotel industry)	Quantitative/Multiple regression Analysis	Positive Emotion	Survey: The questionnaire was developed by using a literature review and in-depth interviews with 8 experts.	*International Journal of Hospitality Management*	No significant influence between employee’s intrinsic motivation to innovate with innovative suggestions in hospitality firms.
2022	Koroglu, Ş., & Ozmen, O.	Turkey/Service industry (Service Banking, retail, healthcare, hospitality and government personnel)	Quantitative/SEM	Engagement	Work Engagement: Utrecht WE scale. IWB: Scott and Bruce(1994)	*Asia-Pacific Journal of Business Administration*	Innovative work behaviour variable was positively affected by the work engagement variable.
2022	Moreno Cunha, A., Marques, C. S., & Santos, G.	European Union Countries (Portugal)/Service industry (Healthcare)	Quantitative/PLS-SEM	Engagement	Work Engagement:UWES-9 (Utrecht Work Engagement Scale (Schaufeli, Bakker, & Salanova, 2006)	*Sustainability*	Findings reveal that work engagement has a significant effect on innovative behaviour.
2022	Batistič, S., Kenda, R., Premru, M., & Černe, M.	European Union Countries/Education industry	Mixed-method research/Correlation and Descriptive statistics	Relationship	Attachement Style: Škerlavaj et al. (2014)	*European Management Journal*	Secure attachment style yielded the most positive results for idea generation and implementation.
2022	Banmairuroy, W.,Kritjaroen, T., &Homsombat, W.	Thailand/Other industry (S-Curve industries)	Quantitative/SEM	Relationship	Knowledge-oriented leader relationship: Weiner (2015)	*Asia Pacific Management Review*	Proper leadership relationship does affects organizational innovation component factor.
2022	Aboramadan, M., Dahleez, K. A., & Farao, C.	Palestine/Education industry.	Quantitative/SEM	Relationship	Inclusive relationship: Carmeli et al. (2010)	*International Journal of Educational Management.*	The findings reveal that inclusive relationship exerts a positive effect on extra-role behaviors such as innovative work behaviors) in the Palestinian higher education.
2022	Hunsaker, W. D.	China/Service industry	Quantitative/SEM	Relationship	Spiritual leader relationship: Fry et al. (2005)	*Current Psychology*	The results of the study concluded that employees’ IWB is positively influenced by the effects of supportive relationship with management.
2022	Kim, K.	South Korea/Nor Specified	Quantitative/SEM	Relationship	Relational Leadership Questionnaire (RLQ) Carifio (2010)	*Sustainability*	Supervisors’ relational leadership was positively related to employees’ performance in innovative work behaviors over time.
2022	Jobbehdar Nourafkan, N., Tanova, C., & Gokmenoglu, K. K.	Turkey/Education industry	Quantitative/Confirmatory Factor Analysis	Positive Emotion	Mindfulness Attention Awareness Scale (MAAS): Brown and Ryan (2003)	*Advance Online Publication*	High levels of mindfulness could positively and directly affect employees’ OCB.

### Research design

Among the 37 articles reviewed, 35 articles opted for quantitative research methodologies, while only two articles employed a mixed-methods approach in their study designs. It is a norm when looking for relationships, or correlations between two or more variables, positivist researchers typically use quantitative methods. ^40^ From Figure 5, we can conclude that positive emotion, positive relationship, engagement, meaningful and accomplishment constructs were measured with various instruments. For positive emotion construct, most of the studies adopted instruments from Porath *et al.* ^41^ Meanwhile, the most widely used measure for assessing work engagement was UWES–Utrecht Work Engagement Scale by ^42^ (Schaufeli *et al.*, 2002) (n = 6).

**Figure 2.**
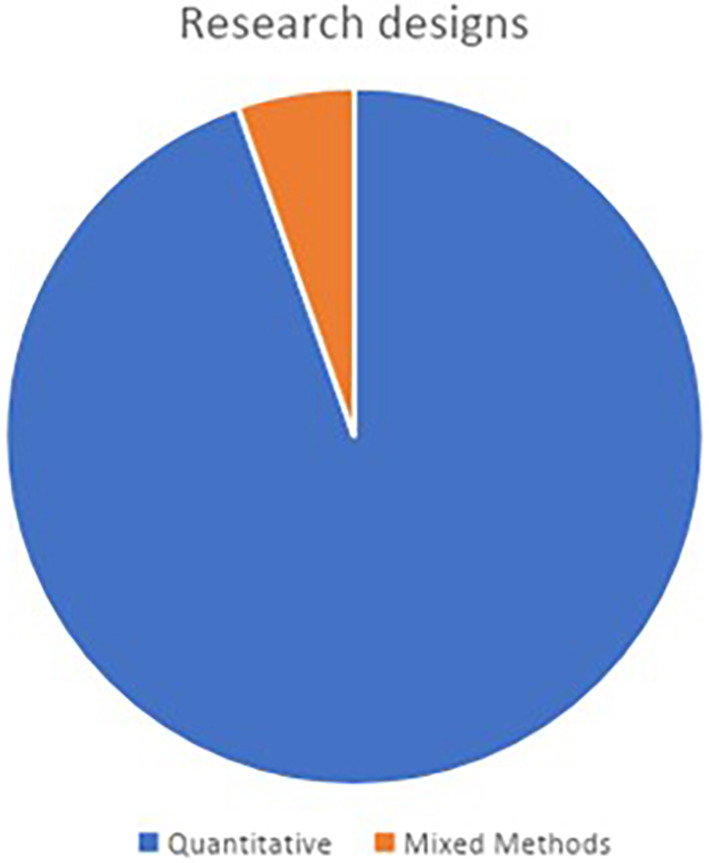
Research designs.

Next, for the positive relationship construct, the majority of researchers used Multi-Factor Leadership Questionnaire (MLQ-5X) by Avolioa *et al.* ^43^ The second commonly most used instrument was the Supportive Relationship Leader from Liden *et al.* ^44^ and Relational Leadership Questionnaire (RLQ) by Carifio. ^44^ On the other hand, Multidimensional Work Motivation Scale was used by Gagné *et al.* ^45^ and direct-consensus, a composition model was used by Chan ^46^ to assess the meaningful factor. Finally, the item used to measure the accomplishment construct was adopted from Reward and Recognition by Spector. ^46^ Structural equation modelling was the most popular technique to analyse the relationship between constructs (n =17). Figure 2 illustrates this highly disproportional distribution of research methodology among the reviewed articles.

### Journal titles and publication volume

There were 37 articles related to PERMA and innovative work behaviour found in 32 journals in various fields as stated in Table 1. There are four articles published in the journal of sustainability between the year 2017 and 2022. ^47^^–^^50^ The authors in these articles discuss the relationship between work engagement and innovative work behaviour. There are two articles published in RAUSP Management Journal. The article written by Dixit and Upadhyay ^13^ has discussed the importance of employee engagement and the significant effect of award and recognition to employee engagement. However, Kundu, Kumar and Lata ^51^ has conducted research on the relationship between intrinsic motivation and innovative work behaviour. There were two articles published by Heliyon. ^52^^,^^53^ The article published in 2020 by Gemeda and Lee ^52^ discussed the effect of engagement and relationship with innovative work behaviour. Sudibjo and Prameswari ^53^ also discussed about the leadership relationship.

**Figure 3.**
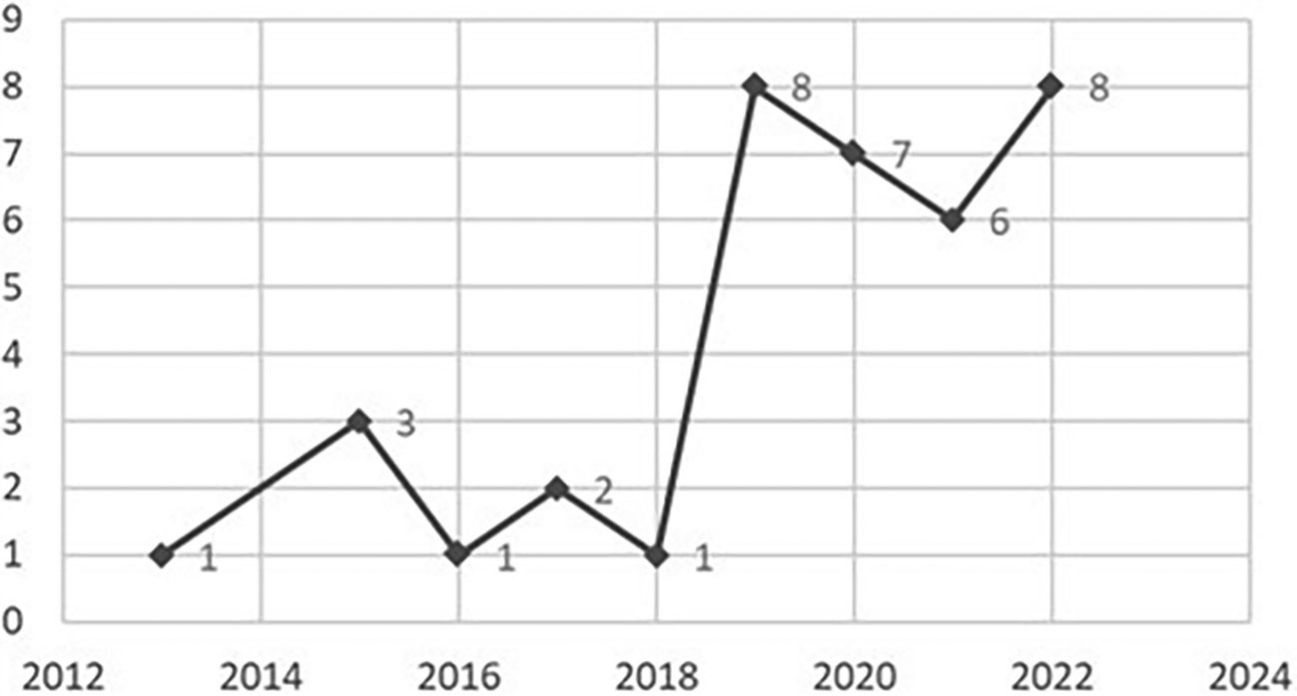
Publication volume by year.

Figure 3 shows an obvious ascending research trend from 2013 to 2022 for PERMA factors and IWB. The number of papers spiked up from one paper in 2013 to eight papers in 2019. The earliest empirical study about the PERMA factor positive emotion and IWB was conducted in 2013 by Yesil and Sozbilir. ^54^ Previous scholars have contributed eight papers about PERMA factors in the year 2019 and year 2022. In both years, the most PERMA factors explored by scholars is the relationship factor with a total of 11 papers. ^48^^,^^49^^,^^55^^–^^63^ This is in line with earlier predictions by Anderson *et al.* ^64^ who suggested the need for future research to examine the impact of relationships in the process of creativity and innovation at various levels of analysis. The pattern shows a growing scholarly interest in PERMA factors such as relationships towards IWB.

**Figure 4.**
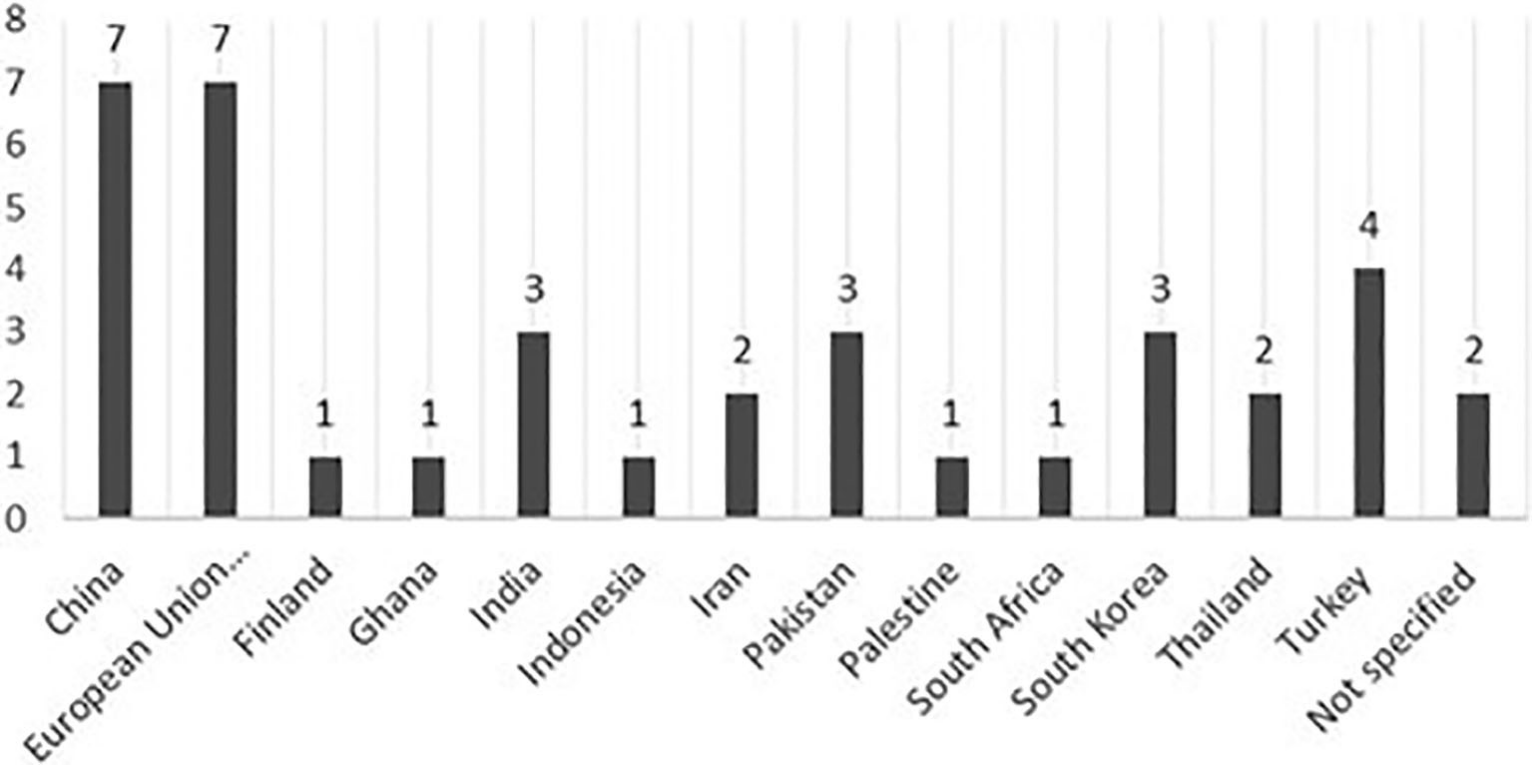
Geographical distribution.

The large geographic distribution in the matrix table shows that PERMA factors are well-known among researchers all around the world. In total, 37 empirical research studies have been published using samples from 13 different countries with China and European Union countries leading the ranking with seven articles, followed by Turkey with four articles, and Pakistan, India and South Korea, each with three articles (see Figure 4 below).

Research scholars from different countries tend to focus on the relationship factor in the PERMA factors. It is the most widely studied PERMA factor as 23 research studies have been published using samples from 12 countries. Engagement is the second most popular PERMA factor being researched with 10 research studies and scholars using samples from seven countries ( *i.e.* European Union countries, India, Iran, South Korea, Thailand and Turkey). The number of research studies performed on the remaining PERMA factor is seven studies for the positive emotion factor, two studies for the meaningful factor, and one study for the accomplishment factor.

What is interesting in Figure 3 is the growth of articles in 2019 to 2020 are contributed by scholars from European Union countries with seven articles altogether. ^47^^,^^58^^,^^61^^,^^62^^,^^65^^–^^67^ The increasing number of papers from European Union countries is contributed by the objective of EU2020 strategy which is to develop the European economy based on research and development (R&D), knowledge and innovation. ^68^

**Table 3.** Journal titles and publication volume.

Journal title	No. of articles found
Administrative Science	1
Advance online publication	1
Arab Economic and Business Journal	1
Asia Pacific Management Review	1
Asian Academy of Management Journal	1
Asia-Pacific Journal of Business Administration	1
Baltic Journal of Management	1
Current Psychology	1
Economic research-Ekonomska istraživanja	1
European Journal of Innovation Management	1
European Management Journal	1
Heliyon	2
Human Systems Management	1
IIMB Management Review	1
International Journal of Educational Management	1
International journal of environmental research and public health	1
International Journal of Environmental Research and Public Health	1
International Journal of Hospitality Management	1
International Journal of Innovation Studies	1
International Journal of Organizational Leadership	1
International Journal of Public Leadership	1
Iranian Journal of Management Studies (IJMS)	1
Iranian Journal of Management Studies (IJMS)	1
Journal of High Technology Management Research	1
Journal of open Innovation	1
Pakistan Journal of Commerce and Social Sciences	1
Procedia Social and Behavioral Sciences	1
Psychology Research and Behavior Management	1
RAUSP Management Journal	2
Revista de Psicología del Trabajo y de las Organizaciones	1
SA Journal of Human Resource Management	1
Sustainability	4

### Research samples

Scholars have shown a significant research interest in the field of PERMA indicating IWB in different types of organisations. For research samples, we found two major industries were surveyed by most of the scholars with eight studies targeted at organisations in the education industries and eight studies targeted at organisations in the service industries. In the education industry, five studies examined the relationship and IWB in an educational setting ^53^^,^^56^^,^^60^^,^^62^^,^^69^: with three studies confirmed that relationship factor has a positive impact on IWB. ^60^^,^^62^^,^^69^ Another three studies in the education industries confirmed a positive relationship between engagement and IWB. ^13^^,^^56^^,^^70^

A total of eight out of 37 papers chose the service sector as their research sample. Among these eight studies, four articles provide empirical evidence regarding the relationship factor and IWB and found a positive relationship between the two variables. ^55^^,^^57^^,^^66^^,^^71^ Two articles provided evidence that IWB in the service sector is positively impacted by engagement. ^47^^,^^72^ Additionally, scholars did not discover a significant link between the positive emotion factor and IWB in the service industry, particularly in the hotel industry. ^3^^,^^54^^,^^67^

With four papers for each, the technology and information technology (IT) industries are the second most researched by scholars. The least surveyed industries for PERMA factors and IWB from 2013 to 2022 were mixed industry, manufacturing industry and other industries. Few scholars did not specify the type of industries that the organisation reside in. For example, Laguna *et al.* ^61^ administered a survey to employees of a small firm that provide private employment and Pukkeeree *et al.* ^73^ chose a human resource officer who was a member of the Personnel Management Association of Thailand as the analysing unit in the study.

### PERMA well-being

*Positive emotion*

The PERMA model of flourishing, introduced by Seligman, ^34^ comprises of five fundamental elements that contribute to psychological well-being such as positive emotions, engagement, relationships, meaning, and accomplishment. The first element of the PERMA well-being model is positive emotions. Emotions are commonly defined as an individual's appraisal of meaning following an experience. ^74^ Positive emotion corresponds to hedonic happiness feelings like pleasure, fun and joy. ^75^^–^^77^ It is good that we feel, and it becomes a primary goal of individuals. Positive emotion helps us to be positive and perceive the environment in a positive manner. ^75^ It also contributes to the expansion of one's horizons and the accumulation of resources. As a result, people can create social and emotional capital by cultivating an open mindset.

Positive emotion also relates to the concept of mental health in that it shows the positive functioning of personal and social life. Some scholars regard positive emotions to be a critical component of psychological well-being. ^78^ Positive emotion is perhaps the only aspect of well-being that has received an extensive study from psychophysiology experts. Numerous research suggest that positive emotions are a main indicator of well-being. It is positively related to life satisfaction, resilience, mindfulness, social rewards, work outcomes and physical health. ^79^

*Engagement*

Seligman ^34^ includes engagement as one of the domains that make up psychological well-being based on the PERMA framework. Engagement is a psychological state in which people report being thoroughly engaged and focused on what they are doing. ^77^ Work engagement can be defined as a positive, fulfilling, work-related state of mind that encompasses vigour, dedication and absorption. ^42^ When it comes to working, vigour refers to a high level of motivation and mental strength and a readiness to put out the effort and persevere in the face of obstacles. Dedication is defined as a solid commitment to one's job and a sense of purpose and enthusiasm, and absorption refers to being completely and pleasantly engaged in one's job to the point where time passes without notice. Various works have used constructs such as employee engagement, work engagement, and role engagement to define work engagement in line with Kahn's conceptualization. ^80^^,^^81^

*Relationship*

With the emerging organization performance trends, the positive work relationships factor between IWB have been explored. ^82^ Positive work relationships factor such as acts of support, kindness, caring, as well as cooperation contribute to well-being of an employee. ^19^^,^^83^ Researchers have started finding out such work relationships that focused on the leader’s ability to attain higher level of performance from their employees. Among several leadership styles, servant leader has a significant impact on the positive workplace which contribute to well-being and IWB. Servant leader prioritise beyond their self-interest and focus on opportunities to help followers grow and develop. ^69^ Opoku *et al.* ^48^ stated that servant leaders focus on the interest and well-being of others and also enhance the motivation of IWB among employees. It shown that those with servant leadership also are able to contribute to the positive work relationship through providing support, direction, opportunity and care which will motivate them to engage in innovative behaviour. ^84^ Indeed, when employees receive support from their organization, it contributes to their positive well-being, which engenders innovativeness in work. ^82^ On the other hand, many scholars cited transformational leadership also in favour of positive work relationships and IWB. ^3^^,^^59^^,^^85^ Transformational leadership clarifies as a strong influencer of employee well-being and IWB because of its motivational impacts by positive relationship between the leader and the subordinates. ^53^ Besides, transformational leaders encourage employees to engage in innovative thinking, problem-solving, and fostering creative behaviour. ^86^ Moreover, transformational leadership promotes a supportive environment where employees are encouraged to take risks, even when there may be negative consequences. ^87^ Therefore, transformational leaders empower employees to build a positive work relationship that support IWB between supervisors and subordinates. Further, inclusive leadership, known as relational leadership, always concern the employees needs and well-being. ^88^ This type of leader always project as supportive behaviour which motivate their employee’s engagement in IWB as they receive emotional and cognition resources. ^60^ The servant leadership, transformational leadership and inclusive leadership confirms that leaders build positive work relationship between employees which foster their well-being. Employees shows high level of energy and commitment to their leaders and thus are more likely to pay back by exhibiting IWB when they received support, care, love, availability and opportunity. ^89^ Thus, a leader is the one who can create positive workplace by flourishing care, support and love of the employees regardless type of leadership styles. ^76^

*Meaningful*

Meaning is a part of the PERMA domain which is considered as a direction to achieve well-being. ^34^ In the literature, meaning tends to refer to believing that a person's life is valuable and that there is a sense of purpose in life that is greater than oneself. ^34^ When people have meaning in life, they will have a purpose that generates passion and motivation. ^90^ Meaning is a superordinate term that encompasses two main dimensions which are comprehension and purpose. ^91^ The first dimension is comprehension, which is the ability to make sense of and comprehend one's life, including one's own self, the external environment, and how one fits into and acts within it. The second dimension is purpose, which is long-term life goals that are self-congruent and inspire relevant action. ^91^ Meaningful refers to engaging in work for the sake of living, not just for a living. ^92^ Cohen-Meitar *et al.* ^93^ posit that meaningfulness in the workplace has a positively related with the employee creativity. As meaning is a crucial component of creative activity, employees who feel their work meaningful ( *e.g.*, those who exhibit higher identified motivation in regard to their work) are more likely to engage in IWB. ^93^ Based on theory of motivation which is self-determination theory (SDT), there are three types of autonomous motivation which are intrinsic motivation, identified motivation and integrated motivation. ^94^ Identified motivation occurs when a person identifies and internalize the perceived value and meaning of behaviour due to its instrumental value. ^45^^,^^94^ Identified motivation is probably connected to IWB. ^95^ Employees who find their work is meaningful will express a higher identified motivation and they are likely to engage in IWB as meaning is an important element of creative behaviour. ^93^

*Accomplishment*

Accomplishment is another PERMA domain that is important for psychological well-being. Accomplishment can be broadly defined as a person's perception of progress toward goals and a sense of accomplishment in their live. ^47^ In order to achieve the goals of task completion, a person needs the ability of competence and efficacy. ^34^ A sense of accomplishment comes from working toward and achieving goals and mastery and efficacy in completing specific tasks. ^18^ Self-efficacy relates to subjective assessments of one's capacity to plan and carry out actions in given conditions, ^96^ while competence refers to accomplishing a specific task to a predetermined standard. ^97^ The accomplishment also can be defined as one's achievement and performance. ^98^ Employee motivation to perform can be increased by intrinsic and extrinsic motivation. ^99^ Examples of extrinsic motivation-enhancing practices are incentives such as economic rewards. ^100^ Janssen ^25^ suggests that employees are willing to be involved in innovative activities beyond contractually stipulated job objectives when their efforts are fairly rewarded in such a social exchange relationship.

## Discussion

### Positive emotion

In this study, there were seven articles have been identified discussing positive emotion and IWB. ^54^^,^^65^^,^^67^^,^^73^^,^^101^^–^^103^ Saether, E. A. ^67^ and González-González, T., and García-Almeida, D. J ^67^ have discussed the intrinsic motivation as a factor for positive emotion under PERMA. The finding of Saether, E. A. ^65^ showed that intrinsic work motivation is positively related to research and development employees’ IWB. However, the research conducted by González-González, T., and García-Almeida, D. J ^67^ found no significant influence between employees’ intrinsic motivation to innovate with innovative suggestions in hospitality firms. Previous research also found that thriving at work has been associated with IWB. ^101^^,^^103^ Thriving at work is a form of positive emotion. The research conducted by Abid, G., Zahra, I., and Ahmed, A. ^103^ has put thriving at work as a mediating mechanism between perceived organization support and innovative work behaviour. The study found that thriving at work is positively related to IWB. Riaz, S., Xu, Y., and Hussain, S. ^54^ discovered that employee thriving was positively associated to organisational support for innovation, which in turn was favourably related to IWB. However, extraversion which was discussed by Yesil, S., and Sozbilir, F. ^54^ in their research also found no significant relation to individual IWB. Research discovered that mindfulness had no direct effect on IWB. ^73^ This link benefits considerably from the mediating role of eudaimonic well-being. The previous research discusses the attainment value as a moderation between engagement and IWB. ^73^ The findings have shown that positive emotion promotes employee engagement which significantly increases IWB.

### Engagement

A total of 10 papers have focused on engagement influence in IWB. ^13^^,^^47^^,^^50^^–^^52^^,^^56^^,^^61^^,^^70^^,^^72^^,^^73^ While most papers measure the direct effect of engagement on IWB, several papers use engagement as a mediator in the study of work behaviour. ^13^^,^^50^^–^^52^^,^^61^^,^^72^ All papers were selected based on the keyword chosen that depicts the engagement domain of the PERMA framework, and the keywords are “work engagement” and “employee engagement”.

This study has found that employee engagement ^13^^,^^51^^,^^56^^,^^70^^,^^73^ and work engagement ^47^^,^^50^^,^^52^^,^^61^^,^^72^ has shown to have a significant relationship with IWB. Ganji *et al.* ^70^ explains the relationship between employee engagement with IWB based on Social Exchange Theory ^104^ in public university in Iran. The study found that the employee engagement has a positive influence on IWB and able to forecast the idea generation, idea promotion and idea implementation in the institution. Dixit and Upadhyay ^13^ investigated the influence of work engagement with IWB based on JD-R model and discovered the work engagement has a significant and positive relationship to IWB. The author found that work engagement mediates the relationship between reward and recognition to IWB. They emphasize on the importance of monetary and non-monetary incentives that will keep the staff engaged and perform innovatively at work. Gemeda and Lee ^52^ suggested that employees who psychologically identify with their work, dedicating and experiencing an emotional connection to their work, seem to be more innovative and put extra effort into completing tasks. Managers also should initiate supporting motivational activities and provide training to their staff to build work engagement. ^73^ Human resource practitioners should consider developing or improving the human resource related policy that supports staff work engagement. This will drive the employee IWB and contribute to a sustainable organisation economic performance. ^50^ The research findings from the papers selected in this study show that the higher the engagement of staff in the work, the higher the staff’s IWB.

### Positive relationship

Having conceptualised positive work relationship practices based on the PERMA model, we structure the results on the relationship between positive work relationship able to influence IWB among employees as supportive organisation and positive relationship between the leaders and employees. ^3^^,^^62^^,^^63^^,^^71^^,^^82^ As such, positive leadership style does influence relationship between leader and employees which influence positively towards IWB. ^49^^,^^59^^,^^66^^,^^105^ Our analysis identified five main leadership styles that could be influence positive work relationship factor which able to encourage IWB among employees. A total of 16 papers have focused on leadership influence positive relationship factor with IWB, and among those 16, four papers confirmed that servant leadership able to promote employees IWB and intrinsic motivation. ^48^^,^^69^^,^^106^^,^^107^ The transformational leadership style had significant positive relationship in employees work engagement and IWB. ^52^^,^^108^ Sudibjo *et al.* ^53^ argues that there is a negative effect on transformational leadership on IWB. On the other hand, two papers examined the role of inclusive leadership in positive relationship between leader and subordinates which positively influence on IWB. ^60^^,^^83^ Only one paper mentioned on authentic leadership predicts the work engagement among employees and in return able to predict employees IWB. ^61^ Hunsaker ^109^ concluded employees IWB is positively influenced by the effects of spiritual leadership which embedded positive workplace relationship. As overall, much less known about how leadership styles influence positive work relationships factor and IWB. The literature calls for further investigation on other antecedents and mediator factors of IWB. ^60^^,^^63^^,^^66^

### Meaningful

In this present study, there are two papers that discuss the relationship between meaning and IWB. ^65^^,^^110^ The papers are selected based on two keywords that represent the meaning domain in PERMA which are “identified motivation” and “spiritual climate”. One of the variables that represent spiritual climate is meaningful. ^111^ Spiritual climate is defined as “the collective perception of the employee about the workplace that facilitates harmony with ‘self’ through meaningful work, transcendence from the limited ‘self’ and operates in harmony with social and natural environment having sense of interconnectedness within it. ^111^ Through the systematic literature review, this study found that meaningful factors represent by a team’s spiritual climate have a positive relationship to team IWB. ^110^ Individuals in business organisations look for the fulfilment of their spiritual needs especially the meaning and purpose of their job and the chance to improve the wider social and ecological environment apart from financial rewards. ^110^ On top of that, a group of employees with a higher spirituality shows a higher propensity to learn and innovate. ^110^ Saether ^65^ who examines the motivational antecedent of employees’ IWB based on self-determination theory (SDT) found that there is a significant and positive relationship between identified motivation and IWB and the relationship is weaker compared to intrinsic motivation. Even though identified motivation has a weaker connection to IWB, Gagné *et al.* ^45^ proposed that it is more practical for organisations to promote identified motivation as it is often easier to encourage employees to internalize the value of their work rather than make the work more fun. Managers may provide constructive and positive feedback to their employees, assign meaningful tasks and give employees the freedom of choice in completing the task to support autonomous motivation. ^112^

### Accomplishment

This study has identified one paper that discuss the relationship between accomplishment and IWB. ^13^ Dixit and Upadhyay ^13^ investigate the effect of job resources such as rewards toward IWB. The paper is selected based on the keyword “reward” which explains the accomplishment domain in PERMA. Dixit and Upadhyay ^13^ conducted a study based on JD-R model to identify the driver of IWB for teachers serving higher education in India. JD-R model was used to analyse how two components of the work environment namely demand, and resources contribute to employee burnout and engagement. ^13^^,^^113^ IWB is an employee extra-role performance that results from work engagement. ^114^^,^^115^ Dixit and Upadhyay ^13^ choose two types of job resources which are job autonomy and reward and recognition in the study and found that rewards were insignificant to IWB. It was also found that rewards and recognition have an indirect impact on IWB through work engagement. ^13^

**Figure 5.**
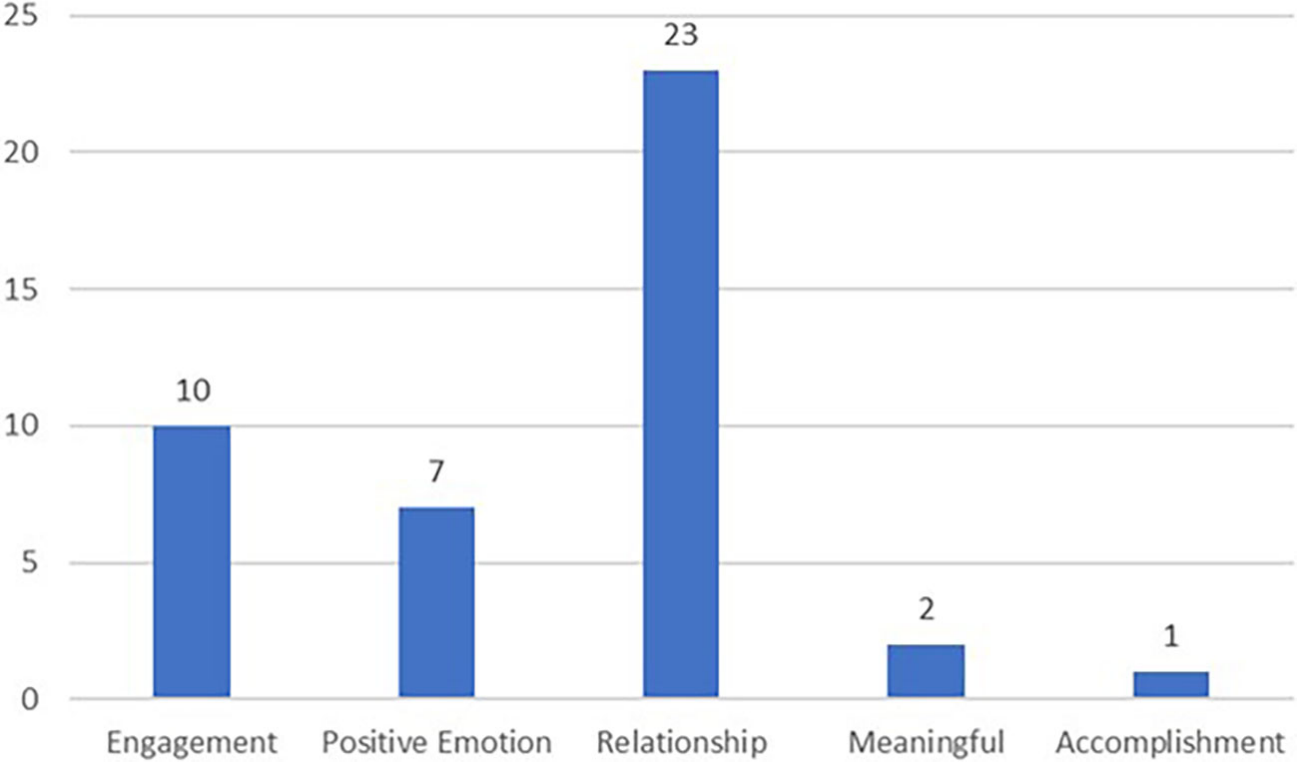
PERMA positive well-being.

All types of PERMA factors that have been studied in the literature are shown in Figure 5. Among the PERMA well-being dimension, positive relationship has been more frequently studied than other types of PERMA well-being from 2012 to 2022. A total of 23 articles studied the effect of positive relationship towards IWB. The least PERMA factors being researched are meaningful and accomplishment.

Meaningful work as a motivating tool may influence people's motivation by allowing them to solve an issue or complete a task that represents their desire to make a difference, which may influence IWB. ^4^^,^^116^ Although studies in intrinsic motivation have discovered a connection between meaningfulness at work and IWB, the relationship between them is not always significant in some studies. ^117^ Latest findings by Wiener *et al.* ^118^ revealed that there is a lack of empirical evidence about how meaningfulness in work influences employees’ IWB. Recent research has underexplored whether meaningful work increases employees' IWB since meaningful work is not vital for clarifying the intrinsic motivation to boost creativity at work. ^119^

Hence, this review draws the same summary that meaningful at work is still an unresolved gap in the literature in terms of examining the relationship with IWB. Meanwhile, there are many research studies that have been conducted on accomplishment towards cooking behaviour, mental illness, well-being, life satisfaction, perfectionism, work environment, leadership and music. ^37^^–^^39^^,^^78^^,^^98^^,^^120^^–^^124^ However, to our knowledge, there is limited previous literature on the relationship between accomplishment and IWB. This is in line with Wammerl *et al.* ^35^ that PERMA well-being represents a quite new well-being model.

**Implications for future practice**

The findings of this review indicate that the existing literature extensively addresses the relationship between five PERMA elements well-being dimensions towards IWB. Even though there is a growing trend of more research on how relationships, engagement, and positive emotions are linked to positive well-being and IWB, there is less research on how the meaningful factor and accomplishment factor are linked to positive well-being and IWB. Hence there is further investigation needed to explore PERMA well-being on IWB. ^124^

Furthermore, according to the empirically supported PERMA theory of positive well-being, ^34^ positive well-being experienced by employees can have a positive organizational impact, including increased productivity, stronger work engagement, improved problem-solving skill and creativity, engaged on innovation and in the long term, more organizational thriving. ^28^^,^^125^^–^^127^

Concluding from the literature, a positive well-being that support IWB in the organisation involved should:

• provide a supportive and cooperative work environment;
• find ways to galvanize employees to feel cheerful and joyful;
• embrace and encourage thriving at work;
• focus on enhancing work engagement among employees;
• be consistent in shaping inclusive and positive relationships at work;
• be savvy at communicating values;
• focus on employees’ intrinsic and extrinsic motivation; and
• grant empowerment to flourish innovativeness.

However, further investigation is still greatly necessary when it comes to the practical implications of positive well-being in a more inclusive and diverse working environment.

Researchers and corporate managers should focus on factors that lead to IWB and explore ways to foster and facilitate employees' creativity. At the organisation level, management team can promote a culture of continuous learning and creativity by encourage employees to seek out new knowledge, embrace failure as a learning experience, and adopt a flexible and adaptive mindset that supports innovation.

For organizations that are determined to flourish employees well-being, the leaders must actively provide the five dimensions of PERMA well-being. Firstly, organizations can foster positivity by celebrating achievements and showing appreciation and care to the employees. This can encourage a positive work environment where employees experience positive emotions, such as happiness, joy, and contentment that boost their overall well-being. Secondly, organizations should create opportunities for employees to be fully engaged and immersed in their work by building positive relationships within the workplace. As sense of belonging and support, leading to higher levels of happiness and satisfaction. By integrating the PERMA theory into their practices, organizations can create a work environment that supports employee well-being and flourishing, leading to increased organisational performance such as higher productivity and innovativeness.

**Recommendation for future research**

This systematic literature review highlights various directions for future research. Firstly, the existing literature reveals numerous aspects of positive well-being in relation to IWB that remain understudied within organizational contexts, including PERMA. This would involve measuring the PERMA elements and IWB over an extended period to understand how changes in well-being factors influence employees' IWB over time. Secondly, from a theoretical development standpoint, it is important to investigate the mediating and moderating factors that may influence the relationship between PERMA elements and IWB. ^117^ For example, exploring the role of intrinsic motivation, such as work engagement in mediating or moderating the relationship can provide a deeper understanding of the mechanisms through which well-being factors promote innovation. Finally, this highlighted that there is an vital need for studies to examine the influence of contextual factors on the relationship between PERMA elements and IWB. Factors such as organizational climate, industry type, organizational size, and cultural differences may impact how the PERMA elements interact with IWB. Exploring these contextual factors can provide insights into the applicability and effectiveness of the PERMA theory across different organizational settings. By exploring these research areas, scholars can deepen their understanding of how the PERMA theory relates to IWB and provide practical insights for organizations seeking to foster innovation through employee well-being.

## Conclusions

The findings of this systematic literature review indicate that between 2012 and 2022, a total of 37 studies focused on PERMA in relation to IWB, primarily conducted in European countries. The majority of these studies employed quantitative research methods (95%) and applied a cross-sectional design. This review utilised 37 articles that meet predefined criteria and constructs a framework exploring the impact of PERMA well-being dimensions on IWB within organizations. The review findings reveal the presence of three influential dimensions that affect employees in IWB, namely: 1) positive emotion, 2) positive relationship, and 3) engagement. However, 4) meaning and 5) accomplishment need further investigation as there are limited studies on those dimensions.

There are certain limitations in this research, including the restricted access to the complete texts of all articles, resulting in the exclusion of some relevant studies on PERMA and IWB within organizations. For future investigations, employing qualitative research methods can provide valuable insights into comprehending the influence of positive well-being on employees' IWB in diverse workplace scenarios.

## Data Availability

All data underlying the results are available as part of the article and no additional source data are required. Figshare: PRISMA flow diagram and checklist for PERMA well-being and Innovative Work Behaviour: a systematic literature review. 10.6084/m9.figshare.24143619. This project contains the following extended data:
-
PRISMA_2020_checklist 14_9_2023.docx -
Figure 1 PRISMA flow diagram.jpg Data are available under the terms of the Creative Commons Attribution 4.0 International license (CC-BY 4.0).
