# Supplementary material for: PERMA well-being and innovative work behaviour : A systematic literature review
Source: F1000Res. 2023 Oct 16;12:1338. [Version 1] doi: 10.12688/f1000research.141629.1 (PMC10751881; doi:10.12688/f1000research.141629.1)
Supplement: Peer review history

# Reviewer report: Reviewer response for version 1

**DOI:** 10.5256/f1000research.155093.r224899

**Published:** 2023-12-18

**Authors:** Ágota Kun

**Affiliations:** 1 Department of Ergonomics and Psychology, Budapest University of Technology and Economics, Budapest, Hungary

The article has set an interesting objective, to examine one of the most popular models within the positive psychology movement, the PERMA model, in the context of innovative behavior within an organization.

 To improve the quality of the article, I suggest the authors to consider the following:

 - I suggest that the PERMA model should not be referred to as PERMA well-being, but as PERMA model of well-being

 - there is no such concept as positive well-being, since well-being is positive in itself so that the adjective 'positive' is superfluous (the opposite of well-being is not negative well-being, but ill-being or unwell)

 - the M factor in the PERMA model is Meaning and not meaningful. meaningful is an adjective, whereas meaning is a noun and this is the original name for the factor - this needs to be corrected in many places in the article

 - in terms of the structure of the paper, I suggest that the PERMA model and its dimensions be presented in detail at the beginning of the article before the literature analysis is presented, as this would be a more logical place

 - I suggest the authors present the relationship between the PERMA elements and IWB in a separate subsection after the literature analysis, because the discussion and presentation of this is presented in the PERMA well-being subsection and the Discussion section, and is therefore rather inconsistent, mixed in both sections and thus somewhat unintelligible and redundant, making the overall understanding of the whole study difficult

 - I would also recommend improving the English of the study

Are the rationale for, and objectives of, the Systematic Review clearly stated?

Yes

Is the statistical analysis and its interpretation appropriate?

Partly

Are sufficient details of the methods and analysis provided to allow replication by others?

Yes

Are the conclusions drawn adequately supported by the results presented in the review?

Partly

Reviewer Expertise:

positive psychology, well-being, workplace happiness, character strengts interventions, mental health at work

I confirm that I have read this submission and believe that I have an appropriate level of expertise to confirm that it is of an acceptable scientific standard, however I have significant reservations, as outlined above.

# Reviewer report: Reviewer response for version 1

**DOI:** 10.5256/f1000research.155093.r224902

**Published:** 2023-11-27

**Authors:** Shahrul Nizam Salahudin

**Affiliations:** 1 Department of Technology Management and Business, Universiti Tun Hussein Onn Malaysia, Batu Pahat, Johor, Malaysia

1. While the abstract mentions a systematic literature review using the PRISMA method, it lacks details about the specific steps involved, such as search terms, databases used, and the inclusion/exclusion criteria. Adding a sentence or two to briefly outline these details would enhance the abstract.
2. The progression of ideas is logical and coherent, providing a smooth transition from the general importance of innovation to the specific focus on the relationship between well-being and innovative work behavior.
3. The conceptualization of well-being and innovative work behavior is clear and aligns with established perspectives. The distinction between hedonic and eudaimonic perspectives and the introduction of the PERMA model contribute to a comprehensive understanding.
4. The breakdown of research samples across industries is beneficial for understanding the scope of the literature. The detailed exploration of industries, such as education and service, and the number of studies within each provides a comprehensive view
5. Overall this is a well written article

Are the rationale for, and objectives of, the Systematic Review clearly stated?

Yes

Is the statistical analysis and its interpretation appropriate?

Yes

Are sufficient details of the methods and analysis provided to allow replication by others?

Yes

Are the conclusions drawn adequately supported by the results presented in the review?

Yes

Reviewer Expertise:

Expert in behavioral sciences

I confirm that I have read this submission and believe that I have an appropriate level of expertise to confirm that it is of an acceptable scientific standard.

# Reviewer report: Reviewer response for version 1

**DOI:** 10.5256/f1000research.155093.r217346

**Published:** 2023-11-14

**Authors:** Imran Saeed

**Affiliations:** 1 The University of Agriculture, Peshawar, Pakistan

Thank you for providing the opportunity to review the manuscript titled "PERMA Well-being and Innovative Work Behaviour: A Systematic Literature Review." Overall, I found this manuscript to be of good quality. However, there is always room for improvement, and I would like to highlight the following points:

1. In the abstract section, the authors did not mention the implications.
2. The introduction lacks a proper rationale and research gap. It is essential to include the latest references in the rationale and research gap.
3. The theoretical background discussed in the manuscript is insufficient. Add some relevant theories and literature to support the theoretical background.
4. In the methods section, the authors mentioned the period from 1st November to 31 December. Why only this period? Is there any valid reason?
5. The rest of the methodology section seems good.
6. Implications should cover practical implications, theoretical implications, and managerial implications.
7. Future recommendations and the conclusion are good.

Are the rationale for, and objectives of, the Systematic Review clearly stated?

No

Is the statistical analysis and its interpretation appropriate?

Yes

Are sufficient details of the methods and analysis provided to allow replication by others?

Yes

Are the conclusions drawn adequately supported by the results presented in the review?

Yes

Reviewer Expertise:

Leadership/Human Resource Management/ Organizational Behavior

I confirm that I have read this submission and believe that I have an appropriate level of expertise to confirm that it is of an acceptable scientific standard, however I have significant reservations, as outlined above.
